# E-Cadherin Downregulation is Mediated by Promoter Methylation in Canine Prostate Cancer

**DOI:** 10.3389/fgene.2019.01242

**Published:** 2019-11-29

**Authors:** Carlos Eduardo Fonseca-Alves, Priscila Emiko Kobayashi, Antonio Fernando Leis-Filho, Patricia de Faria Lainetti, Valeria Grieco, Hellen Kuasne, Silvia Regina Rogatto, Renee Laufer-Amorim

**Affiliations:** ^1^Institute of Health Sciences, Paulista University—UNIP, Bauru, Brazil; ^2^Department of Veterinary Surgery and Anesthesiology, School of Veterinary Medicine and Animal Science, Sao Paulo State University—UNESP, Botucatu, Brazil; ^3^Department of Veterinary Clinic, School of Veterinary Medicine and Animal Science, Sao Paulo State University—UNESP, Botucatu, Brazil; ^4^Department of Veterinary Medicine, Università degli studi di Milano, Milan, Italy; ^5^International Center for Research (CIPE), AC Camargo Cancer Center, Sao Paulo, Brazil; ^6^Department of Clinical Genetics, University Hospital of Southern Denmark, Institute of Regional Health Research, University of Southern Denmark, Vejle, Denmark

**Keywords:** dog, CDH1, prostate, hypermethylation, surface protein

## Abstract

E-cadherin is a transmembrane glycoprotein responsible for cell-to-cell adhesion, and its loss has been associated with metastasis development. Although E-cadherin downregulation was previously reported in canine prostate cancer (PC), the mechanism involved in this process is unclear. It is well established that dogs, besides humans, spontaneously develop PC with high frequency; therefore, canine PC is an interesting model to study human PC. In human PC, *CDH1* methylation has been associated with E-cadherin downregulation. However, no previous studies have described the methylation pattern of *CDH1* promoter in canine PC. Herein, we evaluated the E-cadherin protein and gene expression in canine PC compared to normal tissues. DNA methylation pattern was investigated as a regulatory mechanism of *CDH1* silencing. Our cohort is composed of 20 normal prostates, 20 proliferative inflammatory atrophy (PIA) lesions, 20 PC, and 11 metastases from 60 dogs. The E-cadherin protein expression was assessed by immunohistochemistry and western blotting and gene expression by qPCR. Bisulfite- pyrosequencing assay was performed to investigate the *CDH1* promoter methylation pattern. Membranous E-cadherin expression was observed in all prostatic tissues. A higher number of E-cadherin negative cells was detected more frequently in PC compared to normal and PIA samples. High-grade PC showed a diffuse membranous positive immunostaining. Furthermore, PC patients with a higher number of E-cadherin negative cells presented shorter survival time and higher Gleason scores. Western blotting and qPCR assays confirmed the immunohistochemical results, showing lower E-cadherin protein and gene expression levels in PC compared to normal samples. We identified *CDH1* promoter hypermethylation in PIA and PC samples. An in vitro assay with two canine prostate cancer cells (PC1 and PC2 cell lines) was performed to confirm the methylation as a regulatory mechanism of E-cadherin expression. PC1 cell line presented *CDH1* hypermethylation and after 5-Aza-dC treatment, a decreased *CDH1* methylation and increased gene expression levels were observed. Positive E-cadherin cells were massively found in metastases (mean of 90.6%). In conclusion, low levels of E-cadherin protein, gene downregulation and *CDH1* hypermethylation was detected in canine PC. However, in metastatic foci occur E-cadherin re-expression confirming its relevance in these processes.

## Introduction

Human prostate cancer (PC), the second cause of male cancer-related death in North America, has a variable behavior (Siegel et al., 2019). The mortality rate is associated with metastasis (Huynh et al., 2016), which more commonly affects bone, lymph node, and lung (Siegel et al., 2019). Canine PC is a very aggressive disease associated with high metastatic rate at the diagnosis (more than 85%) being bones, lungs, and iliac lymph nodes, the most common metastatic sites disease-associated (Cornell et al., 2000; Fonseca-Alves et al., 2015a).

Dogs have been reported as a model for human PC and the knowledge regarding molecular aspects of canine PC has increased in recent years (Fonseca-Alves et al., 2018a; Costa et al., 2019; Laufer-Amorim et al., 2019; Rivera-Calderón et al., 2019). These recent studies bring new evidence that canine PC can represent a model to human castration-resistant prostate cancer (CRPC) (Laufer-Amorim et al., 2019). Usually, canine PC lacks NKX3.1, PTEN (Fonseca-Alves et al., 2013; Fonseca-Alves et al., 2018a; Fonseca-Alves et al., 2018b), and androgen receptor expression (Laufer-Amorim et al., 2019) resembling human CRPC. Besides that, canine PC shows alterations in TP53, C-MYC, and MDM2 protein expression (Fonseca-Alves et al., 2013; Fonseca-Alves et al., 2018b). These findings pointed out that the clinical behavior and molecular alterations are similar in both species, making dogs an exciting model in comparative initiatives.

The carcinogenic process, from normal to pre-neoplastic and invasive carcinoma, involves the ability of epithelial cells to detach one another, survive and invade the surrounding tissues (Friedl and Wolf, 2003). Metastasis of PC is a complex process associated with loss of epithelial markers, acquirement of a mesenchymal phenotype, and ability of cells to spread through the lymphatic system or bloodstream (Staník et al., 2014). E-cadherin is a transmembrane protein that has a crucial role in cell adhesion and migration (Debelec-Butuner et al., 2014). E-cadherin also is involved in the β catenin/APC pathway, which is related to cell proliferation and epithelial-mesenchymal transition (EMT) (Tsui et al., 2016). Loss of E-cadherin is associated with poor prognosis in patients with high-grade prostate tumors in both humans (Umbas et al., 1992; Umbas et al., 1994; Abdelrahman et al., 2017; Dhar et al., 2017; Wang et al., 2017; Li et al., 2019) and canine (Fonseca-Alves et al., 2013; Fonseca-Alves et al., 2015a; Kobayashi et al., 2018).

Different mechanisms have been implicated with E-cadherin downregulation in human medicine, including copy number loss (Saramaki and Visakorpi, 2007), somatic mutations (Busch et al., 2017), methylation (Graff et al., 1995; Yoshiura et al., 1995; Li et al., 2001; Mostafavi-Pour et al., 2015), and suppression mediated by ZEB1 and SRC family kinases (Mostafavi-Pour et al., 2015). *CDH1* gene repression promoted by its promoter hypermethylation, plays a crucial role in tumor invasion and spread (Graff et al., 1995; Yoshiura et al., 1995; Li et al., 2001; Mostafavi-Pour et al., 2015). *CDH1* hypermethylation and E-cadherin downregulation have been reported in more than 75% of patients with metastatic PC (Maruyama et al., 2002; Singal et al., 2004; Hoque et al., 2005). Also, *CDH1* promoter methylation is widely studied as a cause of E-cadherin down-regulation in human PC (Graff et al., 1995; Yoshiura et al., 1995; Li et al., 2001; Mostafavi-Pour et al., 2015). However, conflicting results have been reported due to the difficulties in studying methylation (Zhang et al., 2016b). Disparities among methodologies, sample quality, regions of prostatic biopsy, and promoter region evaluated make difficult comparisons among the published studies (Zhang et al., 2016b). Besides that, neoplastic cells can induce hypomethylation and re-express the transcript and its respective protein (Chao et al., 2010), which is compatible with the reversibility phenomenon described in the methylation process.

Transcriptional E-cadherin downregulation mediated by its promoter methylation is widely investigated in human PC (Graff et al., 1995; Yoshiura et al., 1995; Li et al., 2001; Mostafavi-Pour et al., 2015), and E-cadherin plasticity has been proposed during the metastatic progression in human PC (Bae et al., 2011). In high-grade human PC, E-cadherin loss leads to the invasion of metastatic cells to lymph nodes and bones (Putzke et al., 2011). Interestingly, bone metastasis seems to express more E-cadherin than soft tissue metastasis (Putzke et al., 2011). However, few studies evaluating the molecular mechanisms related to *CDH1* silencing have been reported in dogs. Loss of E-cadherin during the lymphatic invasion by neoplastic epithelial cells and E-cadherin re-expression in metastatic foci were previously reported in canine PC (Fonseca-Alves et al., 2015a).

Herein, we investigated E-cadherin gene and protein expression in canine proliferative inflammatory atrophy (PIA), PC and its metastasis as well the methylation status of *CDH1* as a silencing mechanism responsible for the dynamic E-cadherin expression.

## Materials And Methods

### Tissue Selection and Histopathological Evaluation

This cohort is composed of 60 dogs of different breeds, varying from 8 to 14 years old. We selected 20 normal canine prostates, 20 PIA lesions, and 9 PC formalin-fixed embedded-paraffin (FFPE) from the archives from the Department Veterinary Pathology, Sao Paulo State University- UNESP, Brazil. In addition, 11FFPE prostate cancer matched with 11 metastases from the same subjects were selected. All metastases were morphologically analyzed and presented PSA protein expression, as previously described (Fonseca-Alves et al., 2018b). The correspondent fresh frozen tissues from 20 normal canine prostates, 20 PIA lesions, 20 PC samples were used for pyrosequencing and Western blot. All FFPE samples were evaluated by protein and gene expression using immunohistochemistry and qPCR, respectively.

PC samples were collected during surgical or biopsy procedures from animals showing clinical signs. The metastases were identified by imaging tests (X-ray or computed tomography) followed by a biopsy. Normal and PIA samples were collected during necropsies from animals without clinical signs of prostatic disease, with an interval between death and necropsy less than 6 h. All prostate samples were from intact dogs.

The histopathological classification was performed according to the human WHO classification of Tumors of the Urinary System and Male Genital Organs (Humphrey et al., 2016). The Gleason-like system was applied according to Palmieri and Grieco (Palmieri and Grieco, 2015). Briefly, the architectural patterns are evaluated, and the sum of the primary and secondary grades is determined to result in a final Gleason score.

The study was approved by the Animal Ethics Committee according to the national and international guidelines for using animals in research. All animal owners gave written informed consent for the dog’s material, clinical information and examination results to be used for research and academic matters under protocol #107/2015.

### E-cadherin Expression Analysis by Immunohistochemistry

Five-micron thick sections were obtained from FFPE blocks, dewaxed in xylol and rehydrated in graded ethanol. For antigen retrieval, the slides containing the samples were incubated with citrate buffer (pH 6.0) in a pressure cooker (Pascal^®^; Dako, Carpinteria, CA, USA). The samples were then treated with freshly prepared 3% hydrogen peroxide in methanol for 20 min and further washed in Tris-buffered saline. The slides were incubated overnight at 4°C with 0.01µg/µL monoclonal mouse Anti-Human E-cadherin antibody (catalog number GA059, Dako, Carpinteria, CA, USA). A polymer system (catalog number K406511-2, Envision, Dako, Carpinteria, CA, USA) was applied as a secondary antibody conjugated to peroxidase. DAB (3′-diaminobenzidine tetrahydrochloride, Dako, Carpinteria, CA, USA) was used as the chromogen, for 5 min, followed by Harris hematoxylin counterstain. Negative control using mouse universal negative control (Dako, Carpinteria, CA, USA) was included according to the manufacturer’s recommendation. Positive E-cadherin cells in adjacent epithelial cells were considered positive internal controls.

E-cadherin immunoexpression was evaluated according to the number of negative cells. Slides were analyzed under a light microscope (Leica Microsystems, Germany) and 10 images were taken for each slide (Leica QWin V3 software; Leica Microsystems, Germany) at high-power (40X objective) field. Representative areas were qualitatively selected for immunostaining analysis. We choose areas with minimal inflammatory cells, necrosis or connective tissue and with lower E-cadherin staining. Samples were scored based on an assessment of the number of negative cells per the total of cells in 10 high power fields (HPF), according to Hong et al. (2011). These results were expressed in a percentage of negative cells.

### E-cadherin/Ki67 Double Immunostaining

E-cadherin and Ki67 double immunoexpression were performed to exclude cell proliferation as a mechanism associated with E-cadherin focal loss. The procedures were performed as previously reported (Fonseca-Alves et al., 2015b). Briefly, the paraffin sections were deparaffinated in xylol for 15 min and antigen retravel was performed using citrate buffer pH 6.0 solution in a pressure cooker (Pascal, Dako, Carpinteria, CA, USA). Then, endogenous peroxidase was blocked using 8% of hydrogen peroxidase (Dinamica, São Paulo, SP, Brazil), diluted in methanol (Dinamica, São Paulo, SP, Brazil). We used 0.02µg/µL of mouse monoclonal anti-Ki67 antibody (catalog number GA62661-2, Dako, Carpinteria, CA, USA) overnight at 4°C. The polymer system was applied as a secondary antibody for 1 h (catalog number K406511-2, Envision, Dako, Carpinteria, CA, USA) and 3′-diaminobenzidine tetrahydrochloride (DAB, Dako, Carpinteria, CA, USA) was used as the chromogen, for 5 min. The tissue sections were washed with immunohistochemistry buffer (Dako, Carpinteria, CA, USA) and 0.01µg/µL of mouse monoclonal anti-E-cadherin antibody (catalog number GA059, Dako, Carpinteria, CA, USA) was applied overnight at 4°C. After, the HRP magenta chromogen (catalog number GV925, Dako, Carpinteria, CA, USA) was used for 5 min and counterstained with Harris hematoxylin. The positive and negative controls were performed, as described above.

### Immunoblotting

Western blotting was performed to quantify E-cadherin protein expression in seven normal prostates, seven PIA lesions, and seven PC. The frozen prostate samples were sectioned in a cryostat and re-analyzed to confirm the previous diagnosis. The samples were mechanically homogenized, prepared and transferred to nitrocellulose membranes, as previously described (Rivera-Calderón et al., 2016). The blots were blocked with 6% skimmed milk in TBS-T (BioRad, Hercules, CA, USA) for 2 h. Next, the Mouse monoclonal anti-human E-cadherin (0.002µg/µL; catalog number GA059, Dako, Carpinteria, CA, USA) antibody was applied and the slides were incubated at 4°C for 18 h. Goat polyclonal anti-β-actin antibody (0.001µg/µL, catalog number sc-1615, Santa Cruz Biotechnology, Santa Cruz, CA, USA) was used as a loading control. After incubation with the corresponding horseradish peroxidase-conjugated sheep anti-mouse (catalog number NA931, GE Healthcare, Chicago, IL, USA) and donkey anti-goat (catalog number NA9340, GE Healthcare, Chicago, IL, USA) secondary antibodies (0.001µg/µL), the blots were detected by means of chemiluminescence (Amersham ECL Select Western Blotting Detection Reagent, GE Healthcare). Protein bands were quantified by densitometry analysis (Imagequant LAS 500, GE Healthcare, Chicago, IL, USA) and expressed as integrated optical density (IOD). E-cadherin protein expression was normalized using the β-actin values. Normalized data were expressed in means and standard deviations (SD).

### Tumor-Derived Cell Cultures

Two cell lines (PC1 and PC2) were established in our previous study (Zhang et al., 2016). The PC1 cell line was from a 10-years-old, intact, mixed breed dog with non-metastatic PC (cribriform pattern and Gleason score 10). PC2 cell line was from an 11-year-old, intact, poodle dog with metastatic PC (tumor showed cribriform pattern and Gleason score 10). Both cell lines were cultured (the passage 30) in DMEM medium (Lonza, Basel, Switzerland) containing 10% fetal bovine serum (FBS) (LGC Bio, Cotia, SP, Brazil), 1% of penicillin-streptomycin (Thermo Fischer Scientific, Waltham, MA, USA) and amphotericin B (Thermo Fischer Scientific, Waltham, MA, USA) at 37°C in a humidified atmosphere containing 5% CO2. After reaching a minimum of 80% of confluence, both cell lines were processed to obtain DNA. DNA extraction were also performed in their respective primary tumors (fresh frozen samples) followed by pyrosequencing to evaluate the *CDH1* methylation status.

### Methyl Thiazolyl Tetrazolium (MTT) Assay

The 5-Aza 2′deoxycytidine (5-Aza-dC) toxicity was investigated in canine prostatic cells based on the MTT assay. The IC_50_ values were calculated from the dose-response curves to establish the in vitro dosage that will induce demethylation instead of cell death. We used 96-well plates to grow the cancer cells at a density of 2,500 cells per well. The medium was changed every 48 h, and 5-Aza-dC (Sigma-Aldrich, Saint Louis, MO, USA) was added every 24 h. MTT analysis was performed on day 7. The medium was removed, the cells were washed with 3X PBS, and fresh medium was added in each well followed by incubation at 37°C for 4 h. The medium was removed and 200µL of dimethyl sulfoxide (DMSO) (Sigma-Aldrich, Saint Louis, MO, USA) was added in each well and formazan (Sigma-Aldrich, Saint Louis, MO, USA) was solubilized. The optical density (OD) level was measured at 570 wavelengths. Each treatment was performed in triplicate and the experiment in duplicate. Cell viability was calculated into a percentage.

### *CDH1*Gene Expression

Gene expression analysis was performed in our set of samples and both cell lines prior and after 5-Aza-dC treatment. Macrodissection was performed in normal, PIA, PC, and metastatic samples (FFEP) using 16-gauge needles, as previously described (Hoque et al., 2005). mRNA was extracted using RecoverAll™ Total Nucleic Acid Kit (Ambion, Life Technologies, MA, USA) according to the manufacturer’s instructions. cDNA synthesis was performed using total RNA (Applied Biosystems, Foster City, CA, USA), according to the manufacturer’s recommendations. The primers set for *CDH1* (Gene ID: 442858) (Forward: 5′-CAGCATGGACTCAGAAGACAGAAG-3′ and Reverse: 5′-TTCCGGGCAGCTGATAGG-3′) and *ACTB* (Gene ID: 403580) used as endogenous (ACTB, Forward: 5′-GGCATCCTGACCCTCAAGTA-3′ and Reverse: 5′-CTTCTCCATGTCGTCCCAGT-3′) genes were used for RT-qPCR assays. The reaction was conducted in a total volume of 10 µL containing Power SYBR Green PCR Master Mix (Applied Biosystems; Foster City, CA, USA), 1 µL of cDNA (1:10) and 0.3 µM of each primer pair in triplicate using QuantStudio 12K Flex Thermal Cycler equipment (Applied Biosystems; Foster City, CA, USA). A dissociation curve was included in all experiments to determine the PCR product specificity. Relative gene expression was quantified using the 2^-ΔΔCT^ method (Livak and Schmittgen, 2001).

### 5-Aza-2′-Deoxycytidine Treatment

To investigate if hypermethylation is associated with *CDH1* silencing, we treated the PC cell lines with 5-Aza-dC and compared with untreated cells. As previously established by MTT assay, we added 1µg of 5-Aza-dC to the culture medium every 24 h (due to 5-Aza-dC stability) and for seven days. Treated cells were washed with PBS three times. All procedures were performed in duplicate, according to da Costa Prando, et al. 2011). Subsequently, mRNA and DNA were extracted to perform RT-qPCR and pyrosequencing analysis, respectively.

### Quantitative Bisulfite Pyrosequencing

The pyrosequencing analysis was performed to evaluate the frequency of *CDH1* gene promoter methylation in all frozen tissue samples (20 normal prostates, 20 PIA samples, and 20 PC) and cell lines (prior and after 5-Aza-dC treatment). Prostate samples were sectioned in a cryostat to confirm the diagnosis. The bisulfite conversion of the genomic DNA was performed using EZ DNA Methylation-Gold Kit (Zymo Research Corporation, Irvine, CA, USA). The forward (5′ TTTGGGAAGAGGAGGGGG 3′) and reverse primer (5′ CCCTTCCCCTCTCTCTCTC - BIOTIN 3′) of *CDH1* CpG island (Gene ID: 442858) were amplified by PCR (HotStarTaq Master Mix kit - Qiagen). The pyrosequencing was performed using a sequencing primer (5′ TTTGGGAAGAGGAGGGGG 3′) following the manufacturer’s instructions (PyroMark ID Q96, Qiagen and Biotage, Uppsala, Sweden).

### Statistical Analysis

Statistical analysis was performed using GraphPad Prism v.8.1.0 (GraphPad Software Inc., La Jolla, CA, USA). The column test was performed to evaluate data normality. For statistical purposes, the mean of E-cadherin negative cells was used as a threshold to compare the overall survival between patients with over and lower protein expression. Variance analysis (ANOVA) was applied to compare CDH1 transcript levels among normal, PIA and PC samples. Mann-Whitney test was used to evaluate the association of E-cadherin protein and gene expression between two categorical variables. Correlation among the IHC score and clinical parameters, protein expression and transcript levels were also investigated. Mann-Whitney test was applied to evaluate the differences in the methylation levels among the groups. The samples were grouped according to the Gleason score in “low Gleason score” (Gleason score 6 and 8) and “high Gleason score” (Gleason score 10).

## Results

### Clinical Features

**Figure 1:**
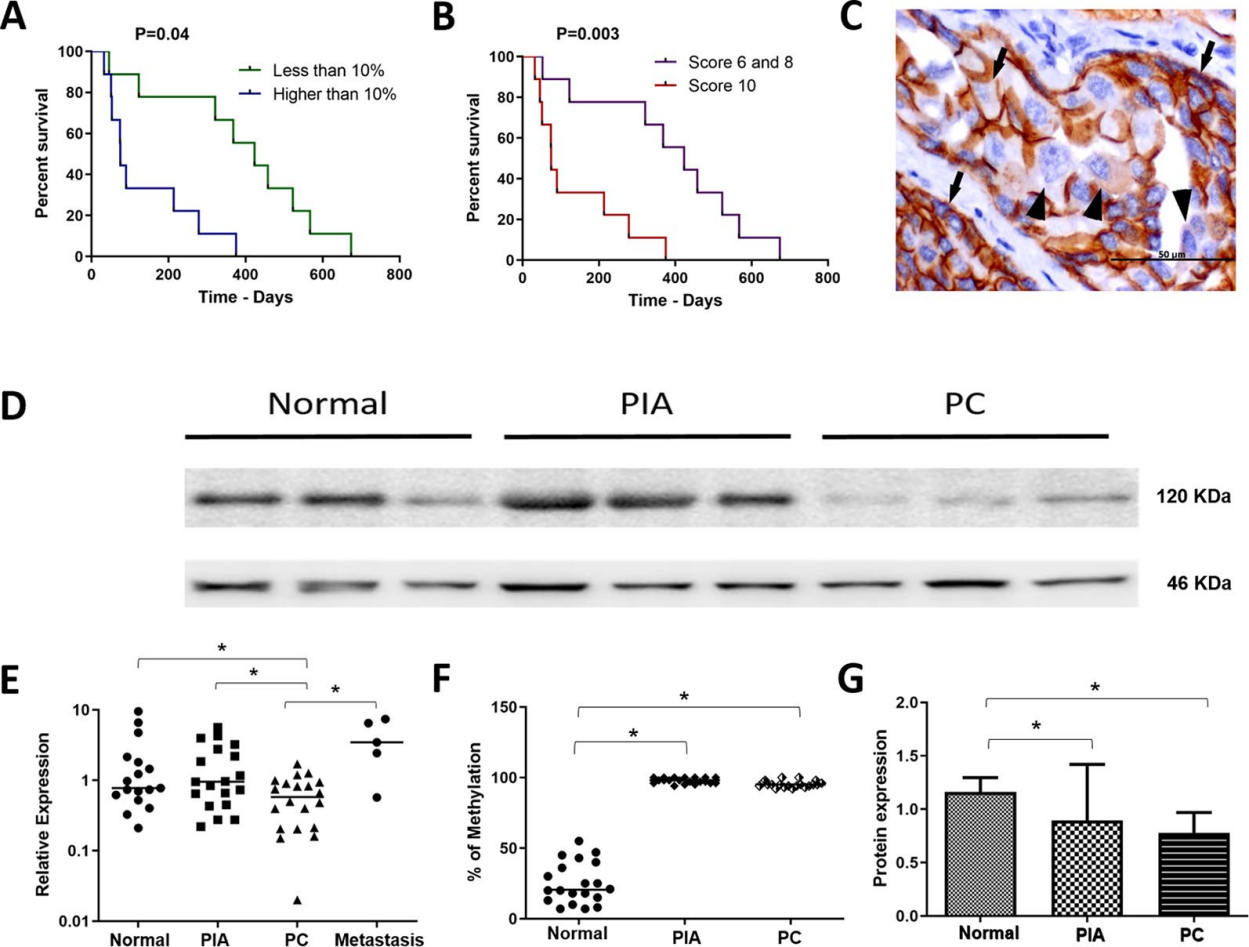
**(A)** survival analysis according to the percentage of E-cadherin negative cells. Patients with over than 10% o E-cadherin negative cells experienced a shorter survival time. **(B)** survival analysis of the canine prostate cancer affected patients according to the Gleason score. Patients with Gleason score 10 experienced a shorter survival time. **(C)** E-cadherin immunohistochemistry showing positive membranous staining (arrows) in neoplastic epithelial cells. Cells were considered E-cadherin negative when partial or total (arrowhead) lack of expression. **(D)** Western blotting showing E-cadherin expression in normal, proliferative inflammatory atrophy and prostate cancer (PC) samples. It is possible to observe E-cadherin down expression in PC samples. **(E)** ANOVA analysis of *CDH1* transcripts in the different canine samples. The prostate cancer (PC) samples showed a lower *CDH1* transcript levels among normal, proliferative inflammatory atrophy (PIA) and metastasis. **(F)** Graphic representation of the percentage of methylation in normal, PIA and PC samples. PIA and PC samples were hypermethylated compared to normal samples. **(G)** graphic representation of E-cadherin protein expression by Western blotting after normalization with β-actin. It is possible to observe lack in both PIA and PC compared to normal samples. *Statistical difference between two variable comparisons.

The clinical features of the 20 PC-affected dogs are described in **Table 1**. Survival information was not available in two of 20 PC patients. The 20 canine PC preseted Gleason scores 6 (30% of cases), 8 (15%) and 10 (55%). Eleven of 20 dogs with PC had metastasis (55%); eight of them (8/11) presented bone and lung metastasis while pelvic bones, intestine and liver were observed in one patient each. From the patients with multiple metastatic sites (bone and lung), only the bone biopsy was evaluated. Seventy-three percent (8/11) of PC patients showing Gleason score 10 had metastasis. Dogs with PC Gleason 8 had no metastasis (n = 3), while 50% (3/6) of cases with Gleason 6 showed metastasis at diagnosis. Patients with lower Gleason score (6 and 8) experienced a higher survival time (P = 0.003) than those with Gleason score 10 (**Figure 1A**).

**Table 1:** Clinical information of 20 canine prostate cancer-affected patients evaluated in this study.

Case	Breed	Age (years)	Histological Pattern	Gleason-like score*	Treatment	Metastasis**	E-cadherin Negative Cells (%)	E-cadherin Positive Cells (%)	Methylation (%)	Follow-up (days)
1	Boxer	14	Cribriform	10	Piroxicam	Lung, Bone and Liver	30	70	94	90
2	Boxer	12	Cribriform	10	LDMT	Bone, Lung	15	85	94	278
3	German Shepherd	8	Small acinar	6	RP	No	2	98	95	453
4	American Cocker Spaniel	9	Small acinar	6	LDMT	No	1	99	95	523
5	Poodle	13	Small acinar	6	N/T	Bone, Lung	10	90	95	321
6	Poodle	14	Small acinar	6	LDMT	No	20	80	94	674
7	Boxer	11	Small acinar	6	Piroxicam	Bone	1	99	92	52
8	MBD	14	Small acinar	6	LDMT	Lung, Intestine	0	100	93	132
9	Poodle	13	Papillary	8	Piroxicam	No	10	90	95	463
10	MBD	12	Papillary	8	Carboplatin + Piroxicam	No	0	100	92	567
11	MBD	11	Papillary	8	LDMT	No	8	92	93	368
12	American Cocker Spaniel	10	Small acinar	10	RP	No	2	98	98	32
13	Poodle	10	Solid	10	RP	No	17	83	100	213
14	MBD	10	Solid	10	Piroxicam	Lung, Liver	0	100	95	55
15	MBD	15	Cribriform with comedonecrosis	10	Doxorrubicin + Piroxicam	Bone, Lung	10	90	94	75
16	American Cocker Spaniel	10	Solid	10	Doxorrubicin	Bone, Lung	22	78	100	78
17	MBD	9	Cribriform	10	LDMT	Bone, Lung	15	85	98	375
18	MBD	7	Cribriform with comedonecrosis	10	N/A	Bone, Lung	25	75	96	N/A
19	MBD	13	Cribriform with comedonecrosis	10	RP	Bone, Lung	5	95	97	45
20	Teckel	11	Cribriform	10	N/A	No	9	91	95	N/A

PC, prostate cancer; MBD, Mixed Breed dog; N/A, Not Available; N/T, No Treatment; RP, Radical Prostatectomy; LDMT, Low-dose metronomic therapy. * Gleason like score was evaluated according to Palmieri and Grieco (2015). ** Metastasis identified at the diagnosis or during the follow-up.

### E-cadherin Immunoexpression

**Figure 2:**
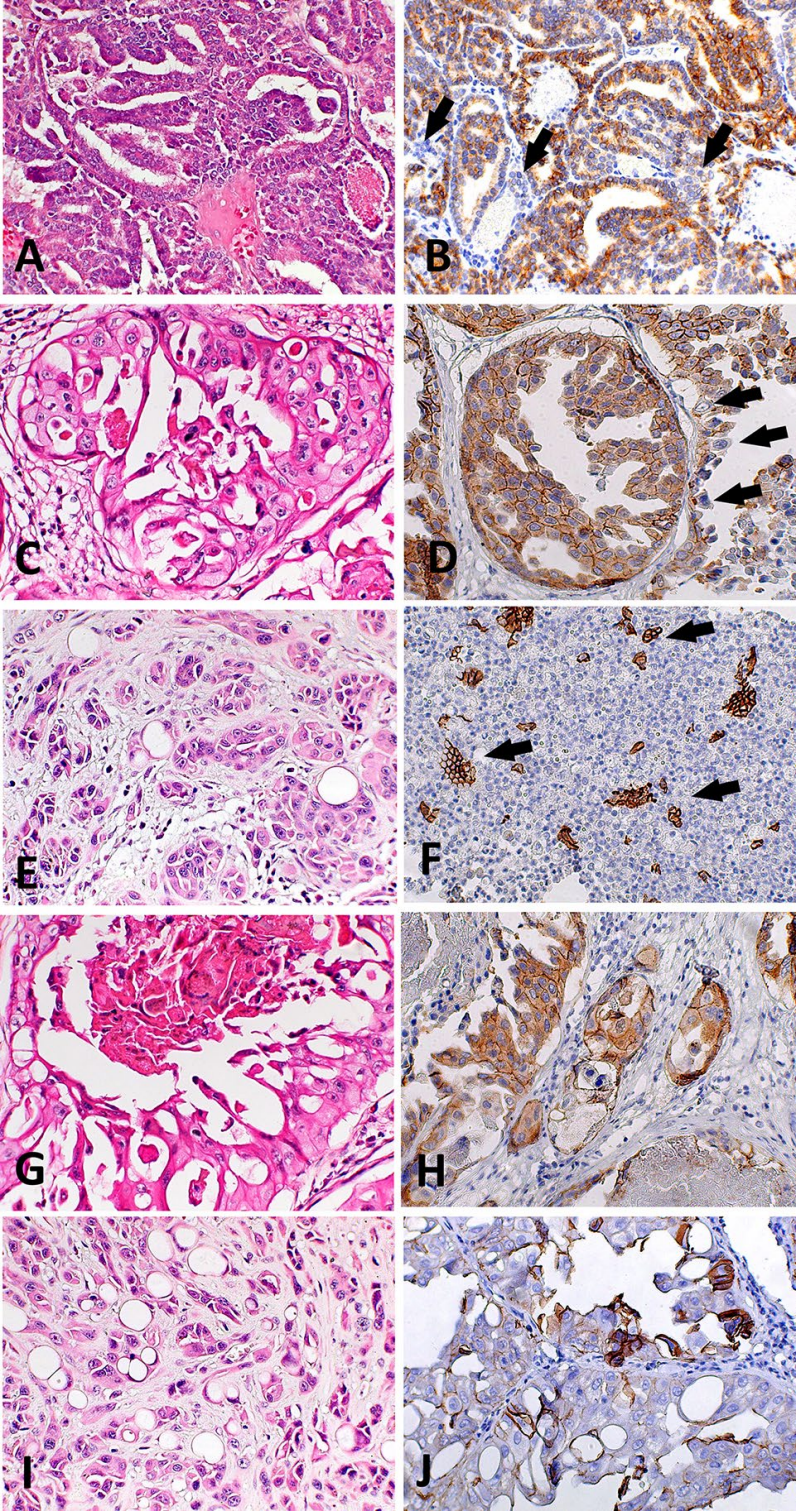
Histological and immunohistochemical E-cadherin evaluation in canine prostate cancer (PC). **(A)** canine PC presenting a papillary pattern. It is possible to observe multifocal areas of E-cadherin loss **(B)** (arrows) in this pattern. **(C)** Canine PC with cribriform patter. Note E-cadherin membranous diffuse expression **(D)** in neoplastic cells and areas of E-cadherin loss (arrows). **(E)** Canine PC with solid pattern. **(F)** area of E-cadherin loss in canine PC with solid pattern. There are only few remaining positive cells (arrows). **(G)** Canine PC showing cribriform with central comedonecrosis pattern. **(H)** is possible to observe membranous E-cadherin expression in neoplastic cells with only few cells showing no E-cadherin expression. **(I)** Canine PC with signet ring pattern. **(J)** It is possible to observe multifocal areas with E-cadherin loss.

We found positive epithelial cells with membranous staining in normal, PIA, PC (**Figure 2**), and metastasis. Cases with less than 10% of negative cells showed a higher survival time (P = 0.004) (**Figure 1B**). A higher number of negative cells was observed in PC (**Figure 1C**) compared to normal and PIA samples. Normal samples showed 100% E-cadherin positive cells; while a mean of 2.1% and 10.5% of negative cells was detected in PIA and PC samples, respectively. Metastases had a mean of 9.5% of negative cells. Tumors showing Gleason score 10 had a higher percentage of negative E-cadherin neoplastic cells compared to PC Gleason scores 6 and 8 and normal samples (P = 0.0003). Metastases had a higher number of negative cells in comparison with normal samples (P = 0.0003) and no statistical difference was observed between all PC samples and metastases (P > 0.05). E-cadherin pattern in each histological subtype is detailed in **Table 1**. The comparison between E-cadherin expression clinical-pathological data is summarized in **Table 2**.

**Table 2:** Mean percentage of E-cadherin negative and positive cells according to the diagnosis and Gleason score.

IHC results	Normal	PIA	PC	Metastasis	Gleason 6	Gleason 8	Gleason 10
Positive cells (%)	100±0	97.9	89.5±4.7	90.5±4.2	98.6±7.3	91%±6.9	82.6±7.1
Negative cells (%)	0±0	2.1	10.5±4.6	9.5±4.2	1.4±7.2	9±6.5	17.4±7.4

IHC’ protein expression by immunohistochemistry; PIA, Proliferative inflammatory atrophy; PC, Prostate cancer.

Comparing the E-cadherin immunoexpression between the primary tumors and its paired metastasis, no statistical difference was found (P > 0.05). The mean of E-cadherin negative cells was similar in primary PC and its paired metastasis (15 ± 7.09 and 16.8 ± 5.25, respectively). No correlation was found between the number of E-cadherin negative cells in the primary PC samples (N = 11) and its respective metastasis (N = 11) (r = 0.076, P = 0.8223). In addition, this comparison was no significant by regression analysis [F (1, 9) = 0.01838, P = 0.08951, *R^2^* = 0.7071]. Although in a limited number of cases, a significant difference was observed comparing E-cadherin negative cells in bone metastasis (N = 9; 14.77 ± 4.02) with those in soft tissues (N = 2; 26 ± 5.0) A positive correlation between E-cadherin negative cells (r = 0.8565, P = 0.0052) was found comparing only primary tumors with the respective paired bone metastasis. We also found a significant regression equation (F (1, 7) = 25.08, P = 0.0016, *R^2^* = 0.7818), comparing primary tumors with their respective metastasis. We observed a positive correlation between the Gleason score and the number of negative E-cadherin neoplastic cells (R = 0.8505 and P < 0.0001) and a significant regression equation [F (1, 18) = 36.18, P < 0.0001), *R^2^* = 0.6678]. Overall, prostate cancer with a high Gleason score showed a higher number of negative E-cadherin cells in comparison with those with lower Gleason scores. The linear regression graphics are shown in **Supplementary Figure 1**.

We also investigated the proliferative index in E-cadherin negative areas using E-cadherin/Ki67 double immunoexpression. All normal samples (N = 20) showed only membranous E-cadherin with no nuclear Ki67 expression. On the other hand, it was identified a higher number of double-stained epithelial cells in PIA samples (N = 20). In PC samples, areas with E-cadherin downregulation showed only scattered Ki67 expression, indicating a low proliferative index (**Supplementary Figure 2**).

### Western Blotting

A strong 120 KDa band was identified in normal prostate tissues (**Figures 1D, G**). No statistical difference was observed comparing the E-cadherin expression in normal prostates with PIA samples. However, a lower E-cadherin expression was detected in PC compared to normal prostate (P = 0.0003) and PIA samples (P = 0.0001). **Supplementary Figure 3** is representative of the Western blotting assays performed in normal prostate, PIA, and PC samples.

### *CDH1*Gene Expression

PC samples showed lower *CDH1* transcript levels in comparison with PIA (P = 0.0038) and normal samples (P = 0.0427) (**Figure 1E**). No statistical difference was observed between the transcript levels in PIA and normal samples. Unfortunately, only five metastatic samples (5/11) were evaluated by RT-qPCR, mainly due to poor mRNA quality. The median of *CDH1* relative quantification (RQ) was 0.7 (0.2–9.5), 0.9 (0.2–5.6), 0.5 (0.02–1.7), and 3.45 (0.6–2.4) in normal, PIA, PC and metastases samples, respectively. In prostate cancer, a strong positive correlation was observed between high levels of E-cadherin protein expression and *CDH1* transcript levels (Spearman R = 0.9429; P = 0.0167) (Significant regression equation: F (1, 4) = 9.654, P= 0.036, *R^2^* = 0.7071). *CDH1* gene expression between the primary tumors (N = 5) and its paired metastasis (N = 5) showed no correlation (r = 0.2000, P = 0.7833) and no significant regression equation [F (1, 2) = 0.06048, P = 0.8216, *R^2^* = 0.01976]. A higher methylation pattern was detected in samples with lower levels of *CDH1* transcripts and a higher number of E-cadherin negative cells, which revealed a direct association of the methylation pattern with gene and protein down expression.

### Quantitative Bisulfite Pyrosequencing

*CDH1* promoter hypermethylation was identified in PIA and PC compared to normal samples (P < 0.0001). The median of methylation was 20.5% (7–55%), 98% (94–100%) and 95% (94–100%) in normal, PIA and PC samples, respectively (**Figure 1F**) (**Supplementary Figure 4**).

### *InVitro* Assays

*CDH1* was hypermethylated and presented lower transcript levels (0.86±0.04) in the PC1 cell line. After the 5-Aza-dC treatment, this cell line presented an inverted methylation pattern and increased gene expression level (1.7 ±0.2).

## Discussion

In this study, E-cadherin gene and protein expression findings were associated with *CDH1* methylation in canine PC, which gives evidence of the regulatory mechanism of *CDH1* in canine PC. E-cadherin is a cell-to-cell adhesion molecule and its loss correlates with epithelial-mesenchymal transition, metastasis and poor prognosis (Putzke et al., 2011; Fonseca-Alves et al., 2015a). Considering the high variation among the different semi-quantitative scores for immunohistochemical evaluation, we counted the number of negative cells and provided a score. We found a higher number of negative E-cadherin cells in PC compared to PIA and normal prostate. Also, a lower number of positive cells was correlated with survival.

Similarly to our findings, Fonseca et al. (2013) and Tsui et al. (2016) reported that PIA presented a lack of E-cadherin expression compared with normal samples. Using Western blot, we confirmed these previous data. Moreover, no statistical difference was observed in PIA compared to PC in cases with a higher E-cadherin expression, which could be explained by the lack of metastatic potential and malignancy of these preneoplastic lesions. Furthermore, during cell proliferation, it is expected the presence of E-cadherin loss by epithelia cells related to cell division instead of a migration (Tsui et al., 2016). For this reason, we performed E-cadherin/Ki67 double staining and confirmed that tumor areas presented E-cadherin losses with no proliferative activity. This result strongly suggests that E-cadherin downregulation is more related to cell migration instead of proliferation. In human PC, *CDH1* hypermethylation and E-cadherin loss is more frequent in metastatic tumors with higher Gleason score (Maruyama et al., 2002). Similar results were observed in our canine PC samples. Although the Gleason score is relatively new in veterinary practice, our study is the first to associate Gleason score with overall survival and E-cadherin downregulation.

In human PC, E-cadherin downregulation is frequent in later stages of the disease and poorly differentiated tumors (Ipekci et al., 2015; Zhang et al., 2016a). Considering the dynamic process of E -cadherin expression, a group of cases with negative cells could also be associated with worse prognosis in canine PC. We showed an association between a higher number of E-cadherin negative cells with shorter survival time, suggesting that the number of E-cadherin negative cells could be used as a prognostic factor. To our knowledge, no previous studies presented the percentage of E-cadherin negative cells and their association with the prognosis in human PC (Graff et al., 1995; Yoshiura et al., 1995; Li et al., 2001; Mostafavi-Pour et al., 2015). On the other hand, in human pancreatic adenocarcinomas, Hong et al. (Hong et al., 2011) described the lowest survival time in patients with total a loss of E-cadherin compared with those with partial loss of the protein expression. The authors suggested that partial and total loss of E-cadherin are an independent negative prognostic factor. In human breast cancer, different authors associated E-cadherin decreased expression with worse prognosis, such as lower overall survival, disease-free interval, positive lymph node (Tang et al., 2012; Ricciardi et al., 2015; Wang et al., 2018), and higher proliferative rate evaluated by Ki-67 (Kashiwagi et al., 2011). In 103 prostate carcinomas, Ipekci et al. (Ipekci et al., 2015) showed E-cadherin decreased expression, but no correlation was found with disease-free survival. The authors suggested that epithelial-mesenchymal transition evaluated by E-cadherin, β-catenin, vimentin and Wnt is a late event in tumor progression. These proteins could not be detected in the primary tumor and, therefore, would not be good predictors of metastasis (Ipekci et al., 2015).

We found a strong positive correlation (r = 0.9424) between E-cadherin protein and gene expression in PC samples. Interestingly, we also found an association between the *CHD1* hypermethylation pattern with gene downregulation. The PC1 cell line was densely hypermethylated and associated with low transcript levels. After 5-Aza-dC treatment, *CDH1* hypomethylation and restoration of gene expression were detected. These results indicated an epigenetic regulation of *CDH1* in canine PC. Similar results were previously described in two prostatic cell lines, DuPro and TSUPr1 (Graff et al., 1995). Considering that DNA methylation is a reversible process, the 5-Aza-dC treatment was efficient in inducing gene demethylation, which suggested that hypermethylated tumors could be sensitive to epigenetic drugs. The hypomethylating agents have been used to treat acute myeloid leukemia (AML) with promising results (Cruijsen et al., 2014). Although our findings are preliminary, dogs could be a preclinical model in precision medicine for testing epigenetic agents in PC patients.

Although cells lacking E-cadherin expression acquire motility and show an invasive and migratory phenotype, only a few cells with no E-cadherin expression are required to develop micrometastasis (Umbas et al., 1994; Canel et al., 2013). Thus, the evaluation of this cell group is relevant for a better understanding of the metastatic process. E-cadherin downregulation occurs in most cases by posttranscriptional mechanisms (Canel et al., 2013). *CDH1* promoter hypermethylation is widely studied in many human cancers, including prostate cancer (Graff et al., 1995; Yoshiura et al., 1995; Li et al., 2001; Mostafavi-Pour et al., 2015). Interestingly, a mean of 90.5% of E-cadherin positive cells was detected in the metastasis. Our data reinforce that the modulation of the metastatic foci and adhesion molecules re-expression are pivotal for the metastasis development (Welch, 2007). A higher number of metastatic cases was observed (N = 3) in patients showing Gleason 10 (N = 8). These samples presented a mean of 17.4% of negative cells. Overall, these results suggest that a group of cells showing lack of E-cadherin expression in primary tumors would have the potential to invade and re-express E-cadherin in metastatic foci.

There is limited information regarding E-cadherin expression in human PC and its paired metastasis (Bae et al., 2011). During the invasion of an artificial basal cell membrane, prostatic cells presented loss of E-cadherin expression and re-expressed after overtaking the membrane (Bae et al., 2011). In dogs, the lack of E-cadherin expression was previously demonstrated in PCs and a complete E-cadherin loss was observed in the neoplastic emboli (Fonseca-Alves et al., 2015a). Interestingly, the paired metastasis showed E-cadherin re-expression. Thus, a dynamic E-cadherin expression occurs during the tumor progression to metastasis. Further studies to evaluate the *CDH1* methylation analysis in circulating prostate cancer cells and its prognostic value could be relevant for clinical purposes.

## Conclusion

Our results suggested an epigenetic regulation of the E-cadherin promoter leading to E-cadherin downregulation in canine PC. The number of negative E-cadherin cells investigated by immunohistochemistry demonstrated the importance of these cells to PC prognosis. Overall, our results indicate that dogs could be a preclinical model for testing hypomethylating agents in precision medicine.

## Data Availability Statement

All datasets generated for this study are included in the article/**Supplementary Material**.

## Ethics Statement

This study was approved by the Animal Ethics Committee of the University of Sao Paulo State, UNESP, Botucatu, Brazil (#107/2015).

## Author Contributions

CF-A wrote the first manuscript draft. CF-A performed the immunohistochemistry and qPCR experiments. CF-A, AL-F, PL, and PK performed the cell culture experiments. CF-A and RL-A conceived the project and grant funding. CF-A, SR, and HK conceived and performed the pyrosequencing experiments. VG contributed constructive comments. RL-A and SR supervised the project and revised the manuscript. All authors read and approved the final version of the manuscript.

## Funding

This research was funded by the Sao Paulo Research Foundation (FAPESP) grant (#2012/18426-1 and 2019/24649-2). National Council for Scientific and Technological Development (CNPq) (#422139/2018-1). We also would like to thank the research grant from the National Council for Scientific and Technological Development (CNPq) (#422139/2018-1).

## Conflict of Interest

The authors declare that the research was conducted in the absence of any commercial or financial relationships that could be construed as a potential conflict of interest.
