# Supplementary material for: E-Cadherin Downregulation is Mediated by Promoter Methylation in Canine Prostate Cancer
Source: Front Genet. 2019 Nov 29;10:1242. doi: 10.3389/fgene.2019.01242 (PMC6895247; doi:10.3389/fgene.2019.01242)
Supplement: Supplementary file 1 [file Image_1.pdf]

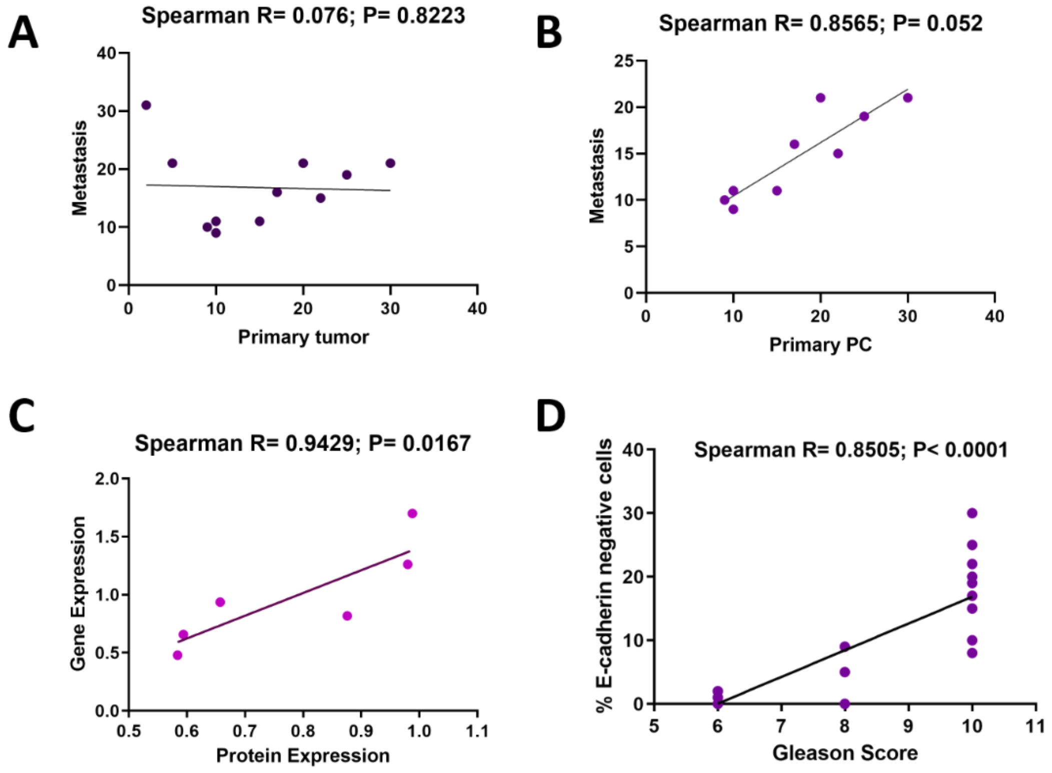

Supplementary Figure 1. Spearman correlation test was applied to compare *CDH1* gene or protein expression with clinical variables. **A**: No significant correlation was found comparing E-cadherin expression in primary tumors versus their paired metastatic lesions. **B**: Positive correlation between the number of E-cadherin negative cells in primary tumors and their paired metastasis. **C**: Positive correlation was detected between *CDH1* expression versus protein expression. **D**: Positive correlation was found between the number of E-cadherin negative cells and Gleason score.
