# Supplementary material for: E-Cadherin Downregulation is Mediated by Promoter Methylation in Canine Prostate Cancer
Source: Front Genet. 2019 Nov 29;10:1242. doi: 10.3389/fgene.2019.01242 (PMC6895247; doi:10.3389/fgene.2019.01242)
Supplement: Supplementary file 2 [file Image_2.pdf]

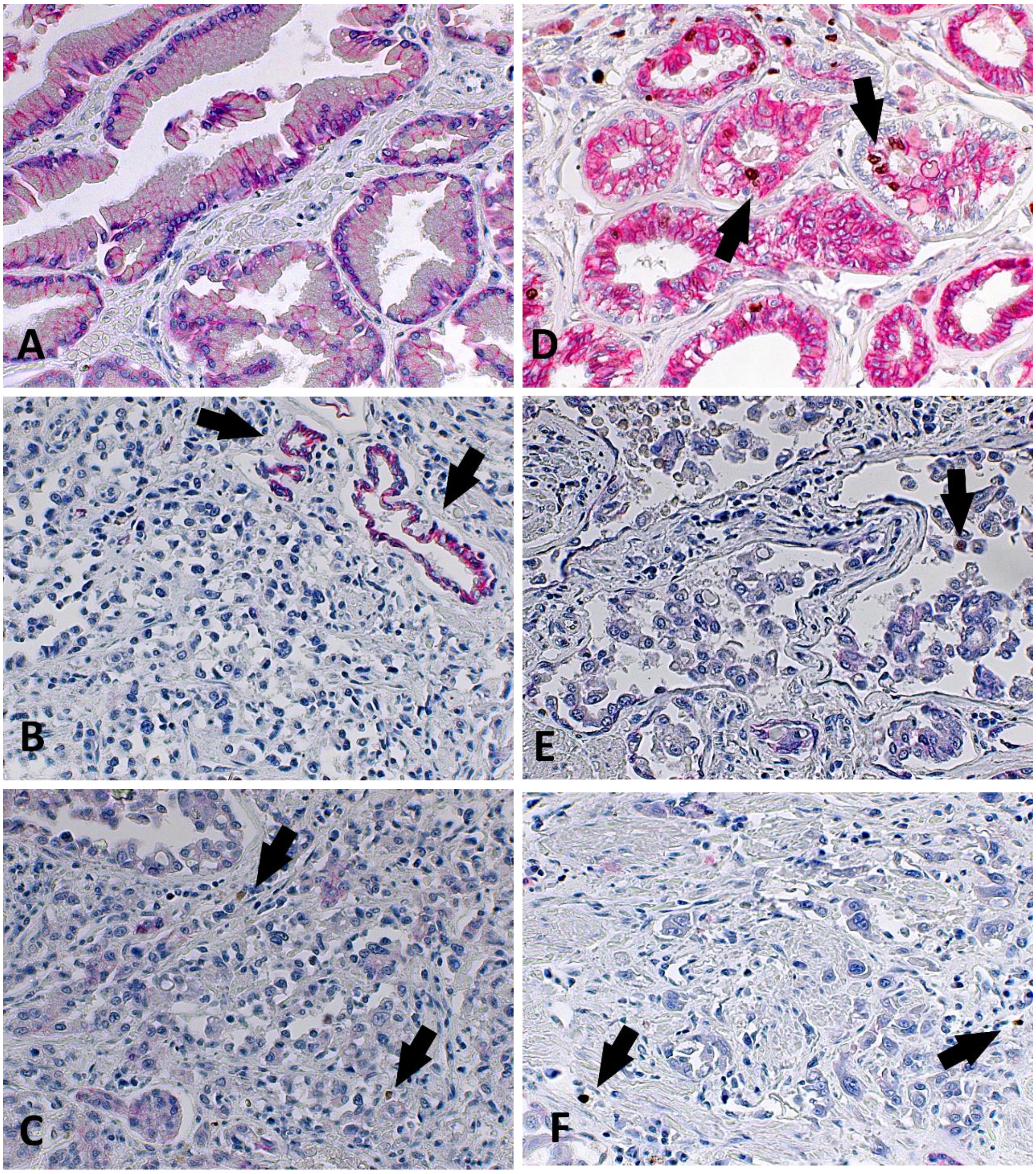

Supplementary Figure 2. E-cadherin/Ki67 double staining in canine prostatic samples. **A**: normal prostatic sample showing membranous E-cadherin expression (pink color) and absence Ki67 immunostaining. **B**: Canine prostate cancer with solid pattern showing adjacent E-cadherin positive gland and no Ki67 expression. **C**: canine prostate cancer with tubular primary pattern, showing E-cadherin loss and few Ki67-positive cells. **D**: double staining in PIA samples showing epithelial cells with membranous E-cadherin expression, stromal cells with Ki67 expression (brown color) and epithelia double stained cells (arrow). **E**: canine prostate cancer with papillary pattern showing no E-cadherin expression and few Ki67 positive cells (arrow), indicating an area of E-cadherin loss with no proliferative activity. **F**: solid canine prostate cancer sample showing different areas of E-cadherin loss, with low Ki67 expression (arrows).

D: double staining in PIA samples showing epithelial cells with membranous E-cadherin expression, stromal cells with Ki67 expression (brown color) and epithelia double stained cells (arrow).
