# Supplementary material for: E-Cadherin Downregulation is Mediated by Promoter Methylation in Canine Prostate Cancer
Source: Front Genet. 2019 Nov 29;10:1242. doi: 10.3389/fgene.2019.01242 (PMC6895247; doi:10.3389/fgene.2019.01242)
Supplement: Supplementary file 3 [file Image_3.pdf]

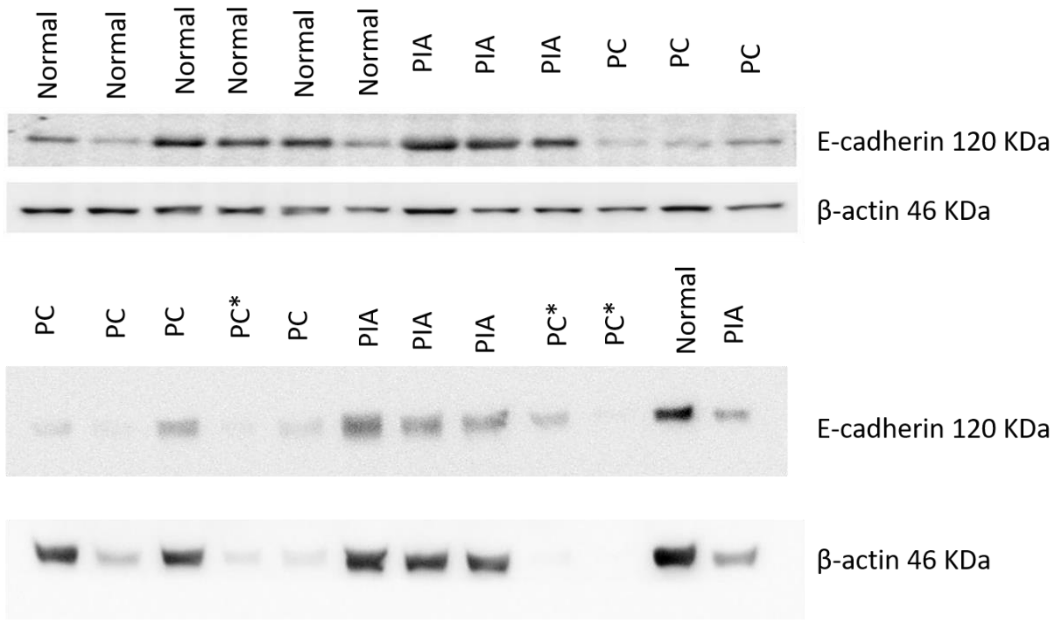

Supplementary Figure 3. Western blot profile of all analysed samples. Samples with asterisk were excluded due lower/absent β-actin expression. PIA: proliferate inflammatory atrophy; PC: prostate cancer.
