# Supplementary material for: E-Cadherin Downregulation is Mediated by Promoter Methylation in Canine Prostate Cancer
Source: Front Genet. 2019 Nov 29;10:1242. doi: 10.3389/fgene.2019.01242 (PMC6895247; doi:10.3389/fgene.2019.01242)
Supplement: Supplementary file 4 [file Image_4.pdf]

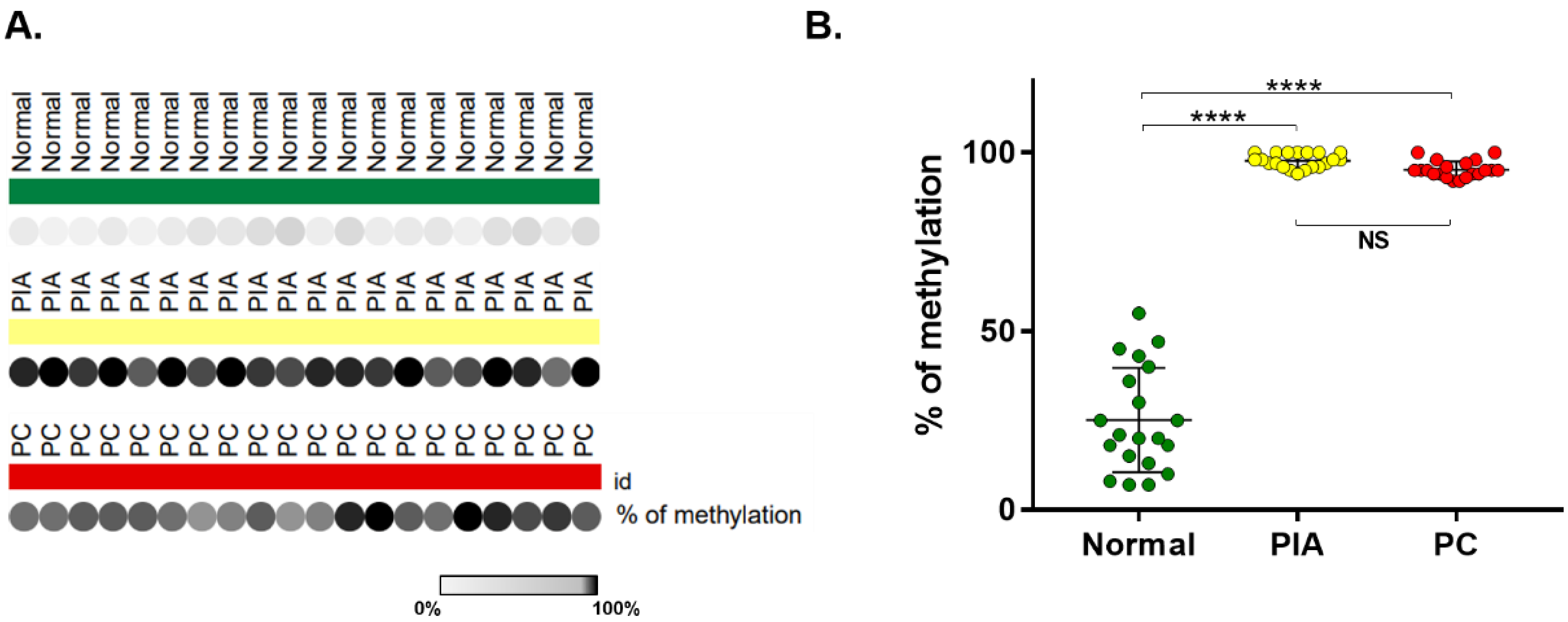

Supplementary Figure 4. Graphics show the methylation levels of CDH1 in Normal, PIA and PC tissues. A. Greyscale heatmap showing a minimum (0 - white) and maximum (100 – black) methylation levels per sample. B. Boxplot representation of the methylation level in Normal, PIA and PC tissues (t-test: ****P < 0.001). Methylation levels are represented by the average of 5 CpGs evaluated in the promoter region of the CDH1 gene. Heatmaps were constructed using Morpheus (https://software.broadinstitute.org/morpheus).
